# Validation of Chemokine Biomarkers in Duchenne Muscular Dystrophy

**DOI:** 10.3390/life11080827

**Published:** 2021-08-13

**Authors:** Michael Ogundele, Jesslyn S. Zhang, Mansi V. Goswami, Marissa L. Barbieri, Utkarsh J. Dang, James S. Novak, Eric P. Hoffman, Kanneboyina Nagaraju, CINRG-DNHS Investigators, Yetrib Hathout

**Affiliations:** 1 Department of Pharmaceutical Science, School of Pharmacy and Pharmaceutical Sciences, Binghamton University—SUNY, Johnson City, NY 13790, USA; mogunde1@binghamton.edu (M.O.); szhan133@binghamton.edu (J.S.Z.); mgoswami@binghamton.edu (M.V.G.); barbieri@binghamton.edu (M.L.B.); ehoffman@binghamton.edu (E.P.H.); nagaraju@binghamton.edu (K.N.); 2 Department of Biomedical Engineering, Binghamton University—SUNY, 4400 Vestal Pkwy E, Binghamton, NY 13902, USA; 3 Department of Health Sciences, Carleton University, Ottawa, ON K1S 5B6, Canada; utkarsh.dang@carleton.ca; 4 Center for Genetic Medicine Research, Children’s Research Institute, Children’s National Hospital, Washington, DC 20010, USA; JNovak@childrensnational.org

**Keywords:** Duchenne muscular dystrophy, Becker muscular dystrophy, disease severity, inflammatory biomarkers, chemokines, validation studies

## Abstract

Duchenne muscular dystrophy (DMD) is a progressive muscle disease involving complex skeletal muscle pathogenesis. The pathogenesis is triggered by sarcolemma instability due to the lack of dystrophin protein expression, leading to Ca^2+^ influx, muscle fiber apoptosis, inflammation, muscle necrosis, and fibrosis. Our lab recently used two high-throughput multiplexing techniques (e.g., SomaScan^®^ aptamer assay and tandem mass tag-(TMT) approach) and identified a series of serum protein biomarkers tied to different pathobiochemical pathways. In this study, we focused on validating the circulating levels of three proinflammatory chemokines (CCL2, CXCL10, and CCL18) that are believed to be involved in an early stage of muscle pathogenesis. We used highly specific and reproducible MSD ELISA assays and examined the association of these chemokines with DMD pathogenesis, age, disease severity, and response to glucocorticoid treatment. As expected, we confirmed that these three chemokines were significantly elevated in serum and muscle samples of DMD patients relative to age-matched healthy controls (*p*-value < 0.05, CCL18 was not significantly altered in muscle samples). These three chemokines were not significantly elevated in Becker muscular dystrophy (BMD) patients, a milder form of dystrophinopathy, when compared in a one-way ANOVA to a control group but remained significantly elevated in the age-matched DMD group (*p* < 0.05). CCL2 and CCL18 but not CXCL10 declined with age in DMD patients, whereas all three chemokines remained unchanged with age in BMD and controls. Only CCL2 showed significant association with time to climb four steps in the DMD group (*r* = 0.48, *p* = 0.038) and neared significant association with patients’ reported outcome in the BMD group (*r* = 0.39, *p* = 0.058). Furthermore, CCL2 was found to be elevated in a serum of the mdx mouse model of DMD, relative to wild-type mouse model. This study suggests that CCL2 might be a suitable candidate biomarker for follow-up studies to demonstrate its physiological significance and clinical utility in DMD.

## 1. Introduction

Duchenne muscular dystrophy (DMD) is an X-linked genetic disease with complex and progressive muscle pathogenesis involving a cascade of altered biological and biochemical events leading to muscle wasting and connective tissue replacement (fibrosis). The pathogenesis is triggered by sarcolemma instability and Ca^2+^ influx due to the lack of dystrophin expression, an essential protein that plays a role in stabilizing the sarcolemma during muscle fiber contraction and relaxation via interaction with dystrophin-associated protein complex [1,2]. Our lab recently generated a large dataset of serum protein biomarkers for DMD using highly multiplexing technologies such as SomaScan^®^ aptamer-based assay (Somalogic, Boulder, CO, USA) [3] and a tandem mass tag (TMT) mass spectrometry-based method [4]. Different groups of biomarkers associated with different pathological pathways of the skeletal muscle were thus identified. These include muscle-centric protein biomarkers reflecting sarcolemma fragility and muscle injury and non-muscle-centric protein biomarkers such as proinflammatory and extracellular matrix remodeling proteins most likely associated with disease pathogenesis and progression. This study focuses on validating and testing three proinflammatory chemokines, CCL2, CCL18, and CXCL10, previously identified by SomaScan^®^ analysis, as being elevated in serum samples of glucocorticoid naïve DMD patients compared to age-matched healthy controls [3]. We hypothesize that chemokines play a role in the early stage of skeletal muscle pathogenesis in DMD, and their circulating levels reflect disease activity.

Chemokines are a group of small secreted proteins involved in chemotaxis and recruitment of immune cells to inflammation sites [5]. They are subdivided based on their primary structures, number, and position of cysteine residues into two major groups: the alpha CXC group, where the two cysteine residues located in the N-terminal end are separated by one amino acid, and the beta CC group, where the two N-terminal cysteine residues are adjacent to each other. Two other minor groups have also been described, the gamma C group and CX3C-d group, but only two and one chemokines discovered under these minor categories to date [6]. All chemokines exert their biological activity by interacting with their respective G-protein coupled receptors and integrins [7]. Proinflammatory chemokines are believed to be involved in the recruitment of different types of immune cells to the site of muscle injury in DMD and other muscle inflammatory diseases [5,8]. However, the role of these recruited immune cells in muscle pathogenesis in DMD remains poorly understood. Some studies have shown that the proinflammatory M1 phenotype macrophages activated in the early stage of the disease are responsible for phagocytosis and muscle fiber damage; this is subsequently replaced by the M2-phenotype macrophages (anti-inflammatory) responsible for muscle regeneration and fibrosis in the later stage of the disease [9]. The recruitment of M1 and M2 macrophages is orchestrated by chemokines. CCL19, CCL21, CCL24, CCL25, CXCL8, CXCL10, and CXCL2 influence the recruitment of the M1 phenotype macrophages, while CCL7 influences the chemotaxis of both M1 and M2 macrophages [10].

In this study, we used a highly specific and reproducible MSD ELISA assay to examine the levels of the three chemokines (CXCL10, CCL2, and CCL18) in serum and muscle samples collected from DMD patients, Becker muscular dystrophy (BMD) patients, and age-matched healthy controls. This approach was also used to investigate the difference in chemokine levels in sera samples of mdx and wild-type mice. The assay was then used to assess the response of these chemokines to glucocorticoid (GC) treatment, their association with age, disease severity, and clinical outcomes. DMD and BMD are two clinically distinct dystrophinopathies. DMD is the most severe form of the disease due to the complete loss of dystrophin expression, while BMD is a milder form due to the partial expression of truncated but functional dystrophin [11,12,13]. Therefore, comparing the levels of circulating chemokines in DMD and BMD will help us understand their association with disease severity and eventually disease progression.

## 2. Materials and Methods

### 2.1. Study Participants and Sample Collection

All specimens used in this study were collected under study protocols approved by the Institutional Review Boards (IRB) at all participating sites. Serum samples from DMD patients and age-matched healthy controls were from a subset of volunteers enrolled in the Cooperative International Neuromuscular Research Group (CINRG) [14]. Participating sites under the CINRG study protocol include the Office of Research IRB administrations at the University of California Davis in Davis, CA, the University of Pittsburgh in Pittsburgh, PA, the Children’s National Hospital in Washington, DC, and the Conjoint Health Research Ethics Board at the University of Calgary in Calgary, Alberta. Informed written consent was obtained from the parents or legal guardians before sample collection. Serum samples from BMD patients were collected remotely by a phlebotomist under a study protocol approved by the Human Subject Research Review Committee at Binghamton University, NY, and shipped to our laboratory for biomarker studies. In the BMD study, following the approval of parental consent/assent, participants were asked to provide a copy of their DNA diagnostic bloodwork to confirm a diagnosis of BMD disease. Additional serum samples from 4-year-old healthy controls were purchased from a third-party company (BIOIVT, Hicksville, NY, USA). Muscle tissue samples from BMD, DMD, and age-matched healthy controls were a gift from Dr. Hoffman’s laboratory previously collected and bio-banked under a study protocol approved by the Regulatory Affairs and Research Compliance administration at the Children’s National Hospital in Washington, DC. All participants consented to their sample being collected for research purposes. Detailed demographics of the patients are listed in Supplemental Table S1. Mouse serum samples were a gift from Dr. Novak (Children’s National Hospital in Washington, DC) and Dr. Nagaraju’s laboratory (Department of Pharmaceutical Sciences (Binghamton University in Binghamton, NY) and were originally collected from 6 to 8-week-old male C57Bl/10J (wild-type) and C57Bl/10J-mdx-23 (mdx-23) mice, respectively. All serum samples were processed with a standardized operating protocol and stored at −80 °C as workable aliquots in polypropylene cryogenic Nalgene vials (Thermo Fisher Scientific, Waltham, MA USA) to avoid repetitive freeze–thaw cycles.

### 2.2. Meso Scale Discovery ELISA Assay of Chemokines

ELISA reagents and plates for measuring the three chemokines CXCL10, CCL2, and CCL18 concentrations were purchased from Meso Scale Diagnostics (Rockville, MD, USA). Analysis of CCL2 and CXCL10 was performed using a U-plex multiplex assay platform for both human and mouse samples, while CCL18 concentrations were measured using an R-plex assay platform. The plates were read using the MSD MESO QuickPlex SQ120 imager, and the values were reported in pg/mL for each target analyte. To test the reproducibility and linearity of the multiplex CXCL10 & CCL2 assay, we compared the results of this assay with the singleplex assay against CCL2 and CXCL10 using longitudinal serum samples of GC naïve DMD patients (*n* = 20). In order to check the reproducibility and stability of the assay, the same samples were analyzed twice on independent days (two weeks apart) by two independent laboratory technicians on the same serum samples from healthy controls (*n* = 10), BMD (*n* = 12) and DMD patients (*n* = 12).

### 2.3. Protein Extraction from Skeletal Muscle Tissue

Muscle tissue samples were lysed in RIPA buffer containing protease inhibitors. RIPA buffer was added to tissue slices at a ratio of 3:1, *v*/*v*. Samples were vigorously vortexed, spun, and kept on ice for 15 min. This action was repeated once, and the samples were kept on ice for 5 min. Samples were then centrifuged at 14,000 rpm for 10 min. Supernatants were collected, and protein concentration was determined using the Pierce^TM^ BCA Protein Assay kit (Thermo Fisher Scientific, Waltham, MA USA). Samples were then aliquoted in workable volumes and stored at −80 °C until analysis. The levels of the three chemokines CXCL10, CCL2, and CCL18, were measured in muscle extract using the same MSD plates and reagents as described above. Data were normalized to total muscle protein in each sample.

### 2.4. Data Processing and Statistical Analysis

The protein concentrations were log-transformed (base 2) and the normality evaluated by the Shapiro–Wilk normality test. A cross-sectional analysis was performed using Student’s *t*-test to compare the levels of the three chemokines (CXCL10, CCL2, and CCL18) in serum and muscle samples of GC naïve DMD patients vs. age-matched healthy controls. Using the same method, we monitored the differences of these chemokines in serum samples of mdx and wild-type mice. 

Then, we conducted a one-way ANOVA to compare the level of the proteins between DMD, control, and BMD in both serum and muscle samples. Tukey’s test for multiple comparisons was used to compare the three groups in a pairwise fashion. Student’s paired *t*-test was conducted on DMD patients pre- and post-GC treatment (*n* = 11) to test for response to GC. Most patients were treated with deflazacort in this study. 

A Pearson correlation coefficient was used to investigate the association of chemokine serum levels with age using cross-sectional samples from DMD, BMD, and healthy controls. Similarly, a correlation was conducted to determine the association of the chemokines with clinical outcome measures and disease severity in DMD and BMD patients, respectively.

A *p*-value < 0.05 was considered statistically significant for the data. All statistical analyses and figures were prepared using GraphPad Prism version 7.0 (GraphPad Software, Inc., San Diego, CA, USA).

## 3. Results

### 3.1. Reproducibility and Stability of the MSD ELISA Assays for Chemokines in Serum Samples

MSD ELISA assays are well-standardized and use validated calibrators and antibodies that undergo rigorous testing and QCs. These QCs include sensitivity, precision, reproducibility, stability of calibrators, with a rigorous cross-reactivity or nonspecific binding cut-off of <0.5% [15]. In this study, we used the following assay kits from MSD: the singleplex U-plex (CXCL10, CCL2), singleplex R-plex assay kits for CCL18, and the U-plex multiplex assay kit (CXCL10 and CCL2 combined). We separately evaluated the singleplex and the multiplex kits for consistency and stability of the assay. A Pearson correlation coefficient was used to check the correlation between experimental runs.

**Figure 1:**
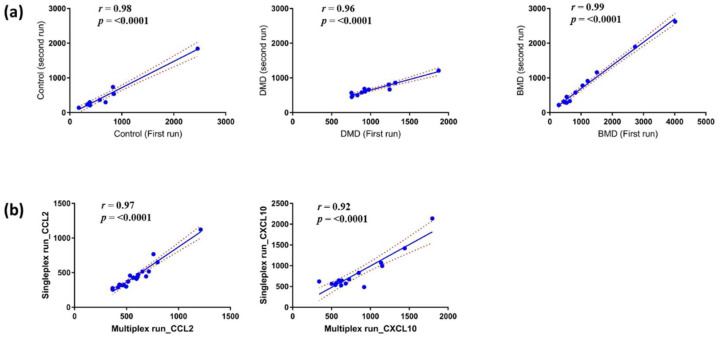
Correlation showing the reproducibility and stability of the singleplex and multiplex assay kits from MSD (*p* < 0.0001). (**a**) Serum samples from control (*n* = 10), DMD, and BMD (*n* = 12) were tested for the stability and reproducibility of the singleplex kit using CXCL10 as reference protein, (**b**) Data obtained from the single run of CCL2 and CXCL10 were plotted against data obtained by the multiplex run of CCL2 and CXCL10.

To assess the repeatability of the assay kit, we conducted two independent experiments with independent operators on two separate days using the same serum samples from DMD patients (*n* =12), BMD patients (*n* = 12), and healthy controls (*n* = 10). The time interval between each experimental run was two weeks, and there was no repetitive freeze–thaw cycle of the serum samples to avoid changes due to sample handling. For each sample, we used a starting serum volume of 10 µL that was diluted six times, and then 25 µL were taken in duplicate from each diluted sample to perform the assay for CXCL10. As shown in Figure 1a, there was a strong correlation between the two runs on independent days by two different operators in all three sample groups (<15% CV, average correlation 0.98) with a *p*-value < 0.0001; this indicates that the method is reliable and consistent for the serum tested. There was no substantial change in CXCL10 trends between each experimental run, and both operators obtained similar results. Similar repeatability between the two operators was obtained for the other two chemokines CCL2 and CCL18, further confirming the robustness of the assay kit. 

We then conducted an independent run to verify if multiplexing affects measurements of a given target. A U-plex 2-plex assay was created for CCL2 and CXCL10. Generally, the R-plex assays are run on separate plates; hence, CCL18 was analyzed separately. Both CCL2 and CXCL10 were multiplexed on a single 96-well plate tested with longitudinal serum samples collected from untreated DMD patients. The results of this experiment were then compared with a singleplex assay of both CCL2 and CXCL10 ran on independent days by two different operators on the same serum samples. The volume and dilution used for this assay are the same as the singleplex method above and were also ran in duplicates. 

The result obtained showed a strong correlation between the multiplex and the singleplex assay kits for CCL2 and CXCL10 (*r* = 0.94, *p*-value < 0.0001); this implies that whether the target was analyzed alone or multiplexed with a second target, similar results were obtained. These results suggest that multiplexing the two chemokines will not affect the outcome of the data (Figure 1b). Moving forward, we multiplexed CXCL10 and CCL2 on the same plate. This action was performed in all the following experiments to evaluate the level of these chemokines in subsequent samples.

### 3.2. Data Validation Using Serum Samples from DMD Patients and Cross-Species Comparison

**Figure 2:**
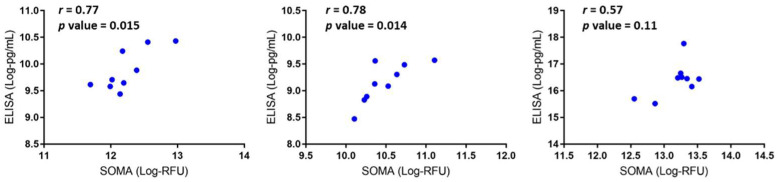
Correlation plots of SomaScan^®^ data vs. ELISA assay data obtained on same DMD serum samples (*n* = 9) for CXCL10 (**left**), CCL2 (**middle**) and CCL18 (**right**).

We previously reported that CCL2, CXCL10, and CCL18 were elevated in DMD (*n* = 18) vs. control (*n* = 12) using SomaScan^®^ [3]. We confirmed these results in a subset of the same DMD patients (*n* = 9) using ELISA and plotted the correlation between the two techniques. As depicted in Figure 2, we found a significant correlation between the two methods for CCL2 and CXCL10. However, there was a statistically insignificant correlation for CCL18. This discrepancy between SomaScan^®^ and MSD ELISA for CCL18 could be due to the small sample size used for the comparative study. Unfortunately, we did not have enough of the same samples used in our previous study to increase the sample size here.

The standardized MSD-ELISA assay above was then tested on a larger sample size of GC naïve DMD patients (*n* = 26) and age-matched healthy controls (*n* = 25) (age range of 4–30 years old). Each sample was run in duplicates, and the CV was generally <15%. The data obtained for each of the tested chemokines is shown under Table 1 with their respective *p*-value and fold change in the DMD group relative to healthy controls. As expected, all three chemokines were significantly elevated in serum samples from DMD patients relative to age-matched healthy controls with a *p*-value < 0.001 for our data.

**Figure 3:**
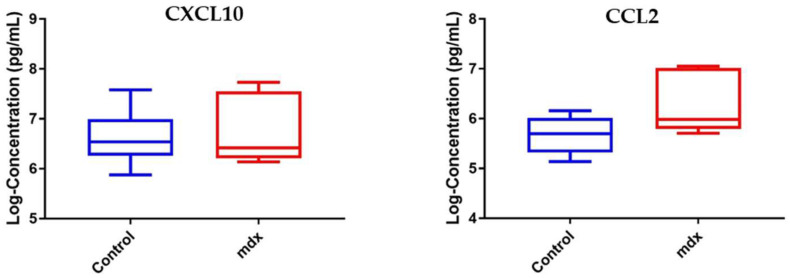
Box plot showing the level of CXCL10 and CCL2 in serum samples of mdx-23 mice (*n* = 10) vs. wild-type BL10 mice (*n* = 14). Left panel CXCL10 remained unchanged between the two groups (*p*-value = 0.6732). Right panel CCL2 was significantly elevated in serum samples of mdx vs. BL10 mice (*p*-value = 0.0046).

To verify if the increased levels of these three chemokines are effectively associated with dystrophin deficiency, we conducted ELISA assays to measure CCL2 and CXCL10 on serum samples collected from mdx-23 mice (*n* = 10) and wild-type mice (*n* = 14). CCL18 was omitted in this experiment because of the unavailability of a CCL18 MSD kit for mice. As shown in Figure 3, only CCL2 was found to be significantly elevated in serum samples of the mdx-23 mice compared to wild-type BL10 mice (Fold change 2.19, *p*-value < 0.05). CXCL10 serum levels were not significantly altered between the two groups studied. 

### 3.3. CCL2 and CXCL10 Are Elevated in Skeletal Muscle Samples of DMD Patients Compared to Healthy Controls

**Figure 4:**
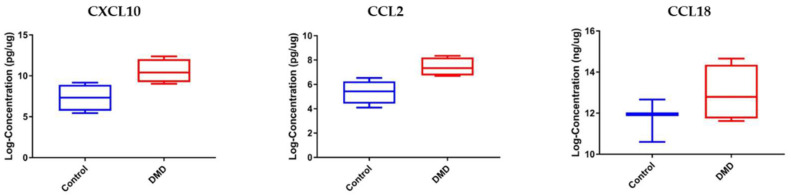
Cross-sectional analysis of chemokine biomarkers in skeletal muscle samples of DMD (*n* = 4) and age-matched healthy controls (*n* = 4). CXCL10 and CCL2 were significantly elevated in DMD vs. control (*p* = 0.026, *p* = 0.0174). CCL18, although elevated in DMD vs. control, was not significant (*p*-value = 0.26). Note: Due to muscle sample limitation from the pediatric population, only three samples were tested for CCL18 in healthy controls.

Levels of the three chemokines studied herein were further examined in skeletal muscle biopsies collected from GC naïve DMD and age-matched healthy control (age 2–8 years old). We hypothesize that chemokines drive DMD pathogenesis and are expected to alter in their levels in the skeletal muscle, the site of injury, and pathogenesis. Due to the limitation of muscle biopsies in pediatrics, we only tested a small number of samples (*n* = 4 per group). As shown in Figure 4, CXCL10 and CCL2 were found to be significantly elevated by a factor of 2.88- and 2.78-fold, respectively, in muscle extracts of DMD patients relative to healthy controls (*p*-value < 0.05). CCL18 was also elevated in DMD muscle relative to healthy control muscle but did not reach statistical significance (Fold change = 2.21, *p*-value = 0.26), probably due to the small sample size. This data agrees with data reported for CCL2 in the skeletal muscle of the mdx mouse model [16] and may indicate a role of this chemokine in recruiting immune cells to the site of muscle injury.

### 3.4. Association of Circulating Chemokines with Age

**Figure 5:**
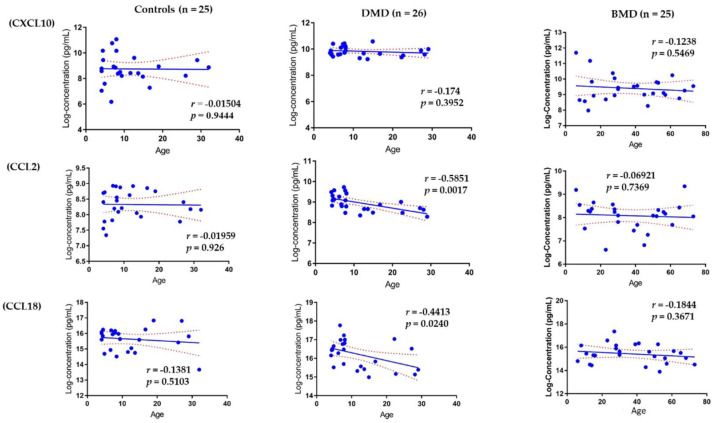
Association of circulating chemokines with age. Left panel: healthy controls (age range 4–32 years old, *n* = 25), middle panel: DMD (age range 4–29 years old, *n* = 26), and right panel is BMD (*n* = 25) for CCL2, CXCL10, and CCL18, respectively. There was no change in CXCL10 in all the samples tested. CCL2 and CCL18 declined over time in DMD patients but remained unchanged in healthy controls and BMD patients.

Age is an important confounding variable in DMD, as most circulating protein biomarkers, especially muscle injury biomarkers, have been shown to decline with age because of the loss of muscle mass [17,18]. As shown in Figure 5, both CCL2 and CCL18 declined with age in DMD patients (age range: 4–29 years old, *n* = 26, *p*-value < 0.05) while CXCL10 remained unchanged over time with age (*n* = 25, *p*-value = 0.3952) in agreement with our earlier study [18]. However, the three chemokines remained unchanged with age in both the BMD group (age range: 6–68 years old, *n* = 25) and in healthy volunteers (age range: 4–32 years old, *n* = 25).

### 3.5. Circulating and Skeletal Muscle Chemokines Are Elevated in DMD Patients Compared to Age-Matched BMD Patients

**Figure 6:**
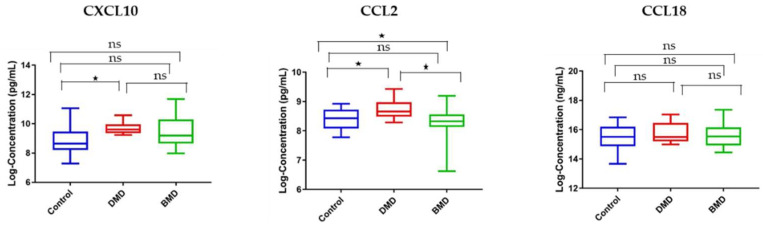
Box plots showing the serum levels of chemokine biomarkers in DMD, BMD, and control. Left panel: CXCL10 serum levels in DMD (*n* = 10) vs. BMD (*n* = 12) vs. healthy control (*n* = 12). Middle panel: CCL2 serum levels in DMD vs. BMD vs. control (*n* = 12). Right panel: CCL18 serum levels in DMD vs. BMD vs. control (*n* = 12). * denotes *p* < 0.05, ns denotes not significant. A one-way ANOVA was used for statistical analysis.

The rationale behind this experiment relies on the fact that BMD is less severe than DMD; hence, the levels of the chemokines may differ between these two diseases and correlate with disease severity. Of the three tested chemokines, only CCL2 was significantly elevated in serum samples of DMD patients compared to age-matched BMD patients (fold change 2.12, *p*-value = 0.024) and age-matched healthy controls (fold change 2.08, *p*-value = 0.03). Although CXCL10 was elevated in the serum of DMD patients compared with BMD patients by 2.03-fold, this difference did not reach statistical difference (*p*-value = 0.0882). CCL18 was not statistically altered in its circulating levels between the three groups with a *p*-value of 0.885 (Figure 6).

**Figure 7:**
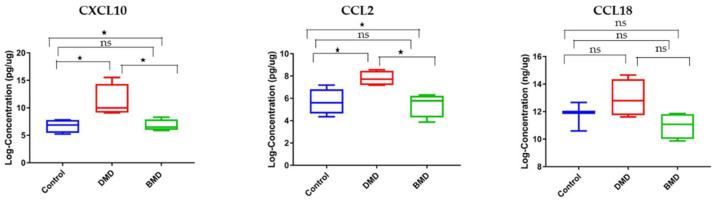
Box plots showing chemokine biomarker levels in skeletal muscle of DMD vs. BMD vs. healthy control. Left panel: CXCL10 in muscle samples of DMD (*n* = 4) vs. BMD (*n* = 4) vs. healthy controls (*n* = 4). Middle panel: CCL2 in muscle samples of DMD (*n* = 4) vs. BMD vs. healthy controls (*n* = 4). Right panel: CCL18 in muscle samples of DMD (*n* = 4) vs. BMD vs. healthy controls (*n* = 3). * denotes *p* < 0.05, ns denotes not significant. A one-way ANOVA was used for statistical analysis.

Consistent with the serum samples, CCL18 levels were not significantly altered in skeletal muscle samples between DMD and BMD (Figure 7). Interestingly, CCL2 was significantly elevated in muscle samples of DMD patients compared with BMD patients (fold change 2.87, *p*-value = 0.02) and age-matched healthy controls (fold change 2.76, *p*-value = 0.03). CXCL10 was also significantly elevated in DMD patients relative to BMD patients, and healthy controls, 3.28- and 3.33-fold, respectively, with a significant *p*-value < 0.05. These results suggest that CCL2 and CXCL10 may be suitable candidates for follow-up studies to define their role in dystrophinopathy pathogenesis.

### 3.6. Response of Circulating Chemokines to Glucocorticoid Treatment

In this study, we sought to examine the response of these three circulating chemokines to glucocorticoids (GCs) treatment. Previously, we found no change in the levels of CXCL10, CCL2, and CCL18 in pre- and post-GC treated patients using the SomaScan^®^ measuring technique [3]. To confirm this finding, the same set of pre- and post-GC treated DMD patients (*n* = 11, age range 4–10 years old) used for the SomaScan^®^ study were tested for longitudinal changes in the circulating levels of CXCL10, CCL2, and CCL18 using the standardized MSD ELISA assay herein. Together, the data obtained for all three chemokines show that they did not respond to GC even after two years of treatment (Table 2). These results agreed with the SomaScan^®^ assay and confirm that the three chemokines are unresponsive to GC treatment.

### 3.7. Association of Circulating Chemokines with Clinical Outcome Measures in DMD Patients and Reported Patient Outcomes in BMD Patients

**Figure 8:**
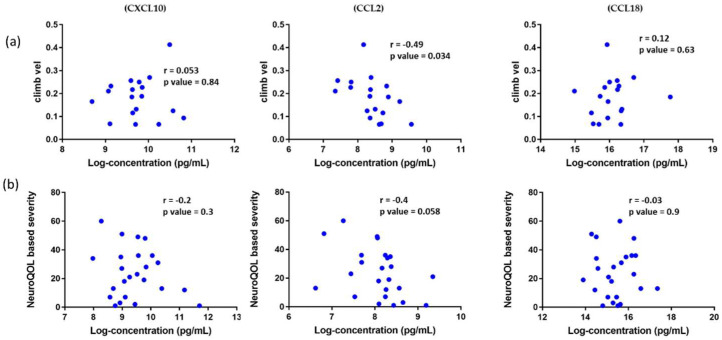
Correlation plots of serum levels for the three tested chemokines with clinical outcome measures: (**a**) correlation with velocity climbing four steps in DMD patients (*n* = 18). Left panel: CXCL10 no correlation; middle panel: CCL2 negatively correlated with climb velocity (*r* = −0.49, *p* = 0.034), and right panel: CCL18 no correlation. (**b**) Correlation with NeuroQOL scores in BMD patients (*n* = 25). Left panel: CXCL10 no correlation; middle panel: CCL2 neared significant negative correlation with NeuroQOL (*r* = −0.4, *p* = 0.058), and right panel: CCL18 no correlation. Pearson correlation coefficient was used.

To examine the association of the three studied chemokines with clinical outcome measures (6 min walk test, run/walk velocity, stand velocity, and climb velocity) we focused on GC naïve DMD patients (4-years-old) to avoid the confounding variable of GC use and age. There was no correlation with clinical outcomes except for CCL2, which negatively correlated with climb velocity and was not adjusted for multiple testing (Figure 8a).

Similarly, we checked the association of the three chemokines with patient-reported outcomes in BMD. Using a subset of BMD patients (*n* = 25, age range 6 to 68), we evaluated the association of circulating CXCL10, CCL2, and CCL18, with the Neuro-QoL^TM^ (Quality of Life in Neurological Disorders)-based clinical severity score used to group the patients based on disease progression, symptoms, and ambulation status. The score ranged from 0 (asymptomatic/very mild) to 64 (most severe). Of all the three chemokines studied, CCL2 was the only chemokine nearing significance with Pearson correlation with a *p*-value of 0.058 (Figure 8b).

## 4. Discussion

Blood accessible biomarkers are becoming highly attractive to use as tools for assessing disease progression and response to therapies, especially in pediatric diseases such as DMD, where other outcome measures remain challenging and labor-intensive for young boys [19]. Although previous high throughput serum proteome profiling to study biomarkers in DMD patients was proven useful for large-scale biomarker discovery [3,4,17,20], a validation step is still required to confirm and test the identified biomarkers. Defining robust biomarker candidates requires a well-defined context of use and an assay that is specific, sensitive, accurate, and reproducible [21]. In this study, we focused on validating three candidate chemokines (CXCL10, CCL2, and CCL18) in DMD and test their association with age, disease severity, GC use, and clinical outcomes. These chemokines were selected because of their relevance in muscle pathogenesis [22,23,24] and the availability of a customized ELISA assay from MSD. The MSD ELISA assays used to measure these three chemokines were found to be specific and stable over time. Furthermore, the multiplexing of two chemokines, CXCL10 and CCL2, under one assay did not affect the accuracy of the data output; this helps reduce analysis time and sample usage. CCL18 was not multiplexed with CCL2 and CXCL10 because R-plex assays often run as a standalone. The intra- and inter-assay experiments had a CV < 15% in agreement with the FDA’s Bioanalytical Method Validation Guidance for Industry [25].

Using the standardized and validated MSD ELISA assay above, we confirmed that CXCL10, CCL2, and CCL18 were significantly elevated in the GC naïve DMD group relative to the healthy control group. Interestingly, CCL2, but not CXCL10, was significantly elevated in serum samples of the mdx mouse model for DMD relative to wild-type mice, further suggesting that increased levels of this circulating CCL2 may be associated with muscle pathogenesis due to dystrophin deficiency. This data agrees with previous studies showing that CCL2 was effectively elevated in the serum of the mdx mouse model [26]. CCL18 was not tested in the mdx mouse model, as there was no validated ELISA assay available for this murine chemokine.

Age is a major confounding variable when confronting biomarker and pathogenesis studies in DMD. We, and others, have shown that many circulating protein biomarkers and especially muscle-centric proteins, decline with age in DMD because of the loss of muscle mass [18,27]. In this study, we further show that CCL2 and CCL18 but not CXCL10 also declined with age in DMD patients. These three chemokines were not affected by age in BMD and healthy controls. The differential effect of age on CCL2 and CCL18 compared with CXCL10 in DMD could suggest a different role for these chemokines in DMD pathogenesis and progression. CCL2 and CCL18 may be involved in chemotaxis and recruit immune cells to the site of muscle injury. Their decline with age in DMD patients could be simply due to the loss of muscle mass and fewer cycles of muscle injury and inflammation.

Although the levels of these studied chemokines seem to be associated with inflammation and disease severity (e.g., DMD vs. BMD), there was no significant correlation with timed function tests in DMD nor patient-reported outcomes (NeuroQOL scores) in BMD for CXCL10 and CCL18. The exception was CCL2, which showed a significant negative correlation with the four steps climbing velocity in GC naïve DMD and neared a significant negative correlation with NeuroQOL in the BMD group, exhibiting a heterogeneous degree of severity; this means that patients with elevated levels of circulating CCL2 fare better in the climb test velocity and NeuroQOL than patients with low levels of circulating CCL2. This data, although intriguing, suggests that elevated CCL2 may have a beneficial effect. This hypothesis is supported by an earlier study conducted in mice showing that CCL2 is required for repairing injured muscle [28]. The role of CCL2, also known as monocyte chemoattractant protein-1 (MCP-1), in the inflammatory processes of muscle injury and repair was reviewed in more detail, and its role in both degeneration and regeneration were discussed [8].

To further link these chemokines to muscle pathogenesis and disease severity, we examined their levels in serum and skeletal muscle samples collected from DMD patients with a severe form of the disease and BMD patients with a milder form of the disease. The rationale behind this experiment is that inflammation leading to muscle pathogenesis is greater in DMD patients than BMD patients, which might be driven by chemokine levels in both serum and muscle samples. Indeed, CCL2, CXCL10, and to some extent CCL18 were found to be significantly elevated in both serum and muscle samples of DMD patients relative to BMD patients and healthy controls. These chemokines were unchanged in serum and skeletal muscle samples of BMD patients relative to age-matched healthy controls. CCL2 exhibited a significant level (~three-fold greater) in skeletal muscle extract of DMD patients compared with BMD and healthy controls; this is further supported by previous studies conducted in animal models showing increased levels of CCL2 in the mdx mouse model in both serum and muscle samples [7,16,26]. It was suggested that CCL2 plays a central role in the recruitment of immune cells at the site of muscle injury, and it could potentially induce the cytotoxic effect of M1 macrophages [7,8,23]. They accomplish this by binding to specific transmembrane receptors, which initiates signals that attract macrophages to the site of damage [8]. A study conducted on the tibialis anterior muscle and diaphragm showed that the mRNA levels of CCL2 and its receptor CCR2 were significantly elevated in the mdx mice model relative to the wild type mice. These expression levels of mRNAs increased with disease progression [23]. Another study conducted on muscle samples of young DMD patients (age 2 years) demonstrated an increase in expression of CCL2 mRNA in this presymptomatic phase [29]; this strengthens the idea that this chemokine is being released at an early stage of the disease and could influence the chemotaxis of infiltrating macrophages in DMD. Although CCL2 is likely involved in chronic inflammation in DMD patients, it has also been reported to play a role in muscle regeneration [7]. Hence, factors such as muscle fiber degeneration, dysregulated regenerative processes, chronic inflammation, and abrogated cell-to-cell communications may affect the levels of circulating CCL2 over time [30]. Therefore, careful studies to define the role of CCL2 in the context of this complex pathogenesis are needed to develop potential therapeutic strategies targeting CCL2 and its receptor [23].

These previous studies on animal models with our data obtained on DMD and BDM patients support our hypothesis that these chemokines, especially CCL2, are likely involved in muscle pathogenesis. However, additional experiments using different muscle diseases and mouse knockout experiments with different inflammation severity are needed to verify this hypothesis.

Interestingly, none of the chemokines responded to GC treatment, confirming our previous SomaScan^®^ data [3]. This observation contrasts with juvenile dermatomyositis patients (JDM), another pediatric inflammatory disease, in which CCL2 and CXCL10 are reported to normalize with treatment to levels similar to healthy controls [31]. This could be due to extensive combination therapies that JDM patients are often treated with.

A significant limitation of this study is the limited number of samples available, especially muscle biopsies. In addition, serum samples and the muscle biopsies reported herein are not from the same subjects, which would have preferably linked the chemokines result from the two specimens to a given patient.

## 5. Conclusions

In summary, we validated three circulating chemokines, CCL2, CCL18, and CXCL10, in DMD serum samples using a reproducible and stable MSD ELISA assay, thereby confirming our previous high-throughput SomaScan^®^ data. CCL2 was especially elevated in the serum of the mdx mouse model and muscle tissue of DMD patients, further supporting its implication in the disease. This data suggests CCL2 as a strong candidate for follow-up studies to define its physiological significance and potential for targeted therapy.

## Figures and Tables

**Table 1:** ELISA assay data obtained for the three tested chemokines on serum samples of DMD patients and age-matched healthy controls.

Protein Name	Cnt (pg/mL)	DMD (pg/mL)	*p*-Value	DMD/Cnt
CXCL10	580 ± 103	942 ± 51	<0.0001	1.62
CCL2	333 ± 21	518 ± 27	<0.0001	1.55
CCL18	57,047 ± 5279	87,746 ± 8936	0.0004	1.54

Data are presented as mean ± standard error, *p*-values are for differences in the mean levels between DMD (*n* = 26) and healthy controls (*n* = 25) and were generated using Student’s *t*-test.

**Table 2:** GC does not affect the level of chemokines pre- and post-treatment.

Protein Name	DMD Pre-Treat (pg/mL)	DMD Post-Treat (pg/mL)	*p* Value
CXCL10	702 ± 118	533 ± 82	0.097
CCL2	449 ± 75	426 ± 64	0.8765
CCL18	64,271 ± 5523	64,856 ± 4690	0.8793

Data are presented as mean ± standard error, *p*-values are for differences in the mean levels between DMD before and after treatment with GC use. Data were generated using paired *t*-test.

## Data Availability

Not applicable.

## Supplementary Materials

The following are available online at https://www.mdpi.com/article/10.3390/life11080827/s1, Table S1: Demographics and ambulation status for DMD and BMD patients whose serum was used for biomarker analysis.

## References

[1] Allen D., Whitehead N.P., Froehner S.C. (2016). Absence of Dystrophin Disrupts Skeletal Muscle Signaling: Roles of Ca^2+^, Reactive Oxygen Species, and Nitric Oxide in the Development of Muscular Dystrophy. Physiol. Rev..

[2] Guiraud S., Davies K.E. (2019). Regenerative biomarkers for Duchenne muscular dystrophy. Neural Regen. Res..

[3] Hathout Y., Liang C., Ogundele M., Xu G., Tawalbeh S.M., Dang U.J., Hoffman E., Gordish-Dressman H., Conklin L.S., Anker J.N.V.D. (2019). Disease-specific and glucocorticoid-responsive serum biomarkers for Duchenne Muscular Dystrophy. Sci. Rep..

[4] Alayi T.D., Tawalbeh S.M., Ogundele M., Smith H.R., Samsel A.M., Barbieri M.L., Hathout Y. (2020). Tandem Mass Tag-Based Serum Proteome Profiling for Biomarker Discovery in Young Duchenne Muscular Dystrophy Boys. ACS Omega.

[5] Luster A.D. (1998). Chemokines—Chemotactic Cytokines That Mediate Inflammation. N. Engl. J. Med..

[6] Zlotnik A., Yoshie O. (2000). Chemokines. Immunity.

[7] De Paepe B., De Bleecker J.L. (2013). Cytokines and Chemokines as Regulators of Skeletal Muscle Inflammation: Presenting the Case of Duchenne Muscular Dystrophy. Mediat. Inflamm..

[8] Deshmane S.L., Kremlev S., Amini S., Sawaya B.E. (2009). Monocyte Chemoattractant Protein-1 (MCP-1): An Overview. J. Interferon Cytokine Res..

[9] Tidball J.G., Welc S.S., Wehling-Henricks M. (2018). Immunobiology of Inherited Muscular Dystrophies. Compr. Physiol..

[10] Xuan W., Qu Q., Zheng B., Xiong S., Fan G.-H. (2014). The chemotaxis of M1 and M2 macrophages is regulated by different chemokines. J. Leukoc. Biol..

[11] Davies K.E. (1997). Challenges in Duchenne muscular dystrophy. Neuromuscul. Disord..

[12] Wilson K., Faelan C., Patterson-Kane J., Rudmann D.G., Moore S., Frank D., Charleston J., Tinsley J., Young G.D., Milici A.J. (2017). Duchenne and Becker Muscular Dystrophies: A Review of Animal Models, Clinical End Points, and Biomarker Quantification. Toxicol. Pathol..

[13] Morales J.A., Mahajan K. Dystrophinopathies. https://www.ncbi.nlm.nih.gov/pubmed/30480978.

[14] McDonald C.M., Henricson E.K., Ms R.T.A., Han J.J., Escolar D.M., Dpt J.M.F., Duong T., Ms A.A., Clemens P.R., Hoffman E. (2013). The cooperative international neuromuscular research group duchenne natural history study-a longitudinal investigation in the era of glucocorticoid therapy: Design of protocol and the methods used. Muscle Nerve.

[15] Ning Q., Rangwala S., Shelburne C., Kobayashi L., Liu P., Lu C., Stewart D., Tsionsky M., Plisova T., Oberoi P. (2015). Development and Characterization of U-PLEX^®^ Cytokine Assays.

[16] Porter J.D., Guo W., Merriam A.P., Khanna S., Cheng G., Zhou X., Andrade F.H., Richmonds C., Kaminski H.J. (2003). Persistent over-expression of specific CC class chemokines correlates with macrophage and T-cell recruitment in mdx skeletal muscle. Neuromuscul. Disord..

[17] Spitali P., Hettne K., Tsonaka R., Charrout M., Bergen J.V.D., Koeks Z., Kan H., Hooijmans M.T., Roos A., Straub V. (2018). Tracking disease progression non-invasively in Duchenne and Becker muscular dystrophies. J. Cachex-Sarcopenia Muscle.

[18] Hathout Y., Brody E., Clemens P.R., Cripe L., DeLisle R.K., Furlong P., Gordish-Dressman H., Hache L., Henricson E., Hoffman E. (2015). Large-scale serum protein biomarker discovery in Duchenne muscular dystrophy. Proc. Natl. Acad. Sci. USA.

[19] Grounds M.D., Terrill J.R., Al-Mshhdani B.A., Duong M.N., Radley-Crabb H.G., Arthur P. (2020). Biomarkers for Duchenne muscular dystrophy: Myonecrosis, inflammation and oxidative stress. Dis. Model. Mech..

[20] Ayoglu B., Chaouch A., Lochmüller H., Politano L., Bertini E., Spitali P., Hiller M., Niks E., Gualandi F., Pontén F. (2014). Affinity proteomics within rare diseases: A BIO—NMD study for blood biomarkers of muscular dystrophies. EMBO Mol. Med..

[21] Szigyarto C.A.-K. (2020). Duchenne Muscular Dystrophy: Recent advances in protein biomarkers and the clinical application. Expert Rev. Proteom..

[22] Wynn T.A. (2007). Cellular and molecular mechanisms of fibrosis. J. Pathol..

[23] Mojumdar K., Liang F., Giordano C., Lemaire C., Danialou G., Okazaki T., Bourdon J., Rafei M., Galipeau J., Divangahi M. (2014). Inflammatory monocytes promote progression of Duchenne muscular dystrophy and can be therapeutically targeted via CCR 2. EMBO Mol. Med..

[24] De Paepe B., Creus K.K., Martin J.-J., De Bleecker J.L. (2012). Upregulation of chemokines and their receptors in duchenne muscular dystrophy: Potential for attenuation of myofiber necrosis. Muscle Nerve.

[25] Kadian N., Raju K.S.R., Rashid M., Malik M.Y., Taneja I., Wahajuddin M. (2016). Comparative assessment of bioanalytical method validation guidelines for pharmaceutical industry. J. Pharm. Biomed. Anal..

[26] Kranig S.A., Tschada R., Braun M., Patry C., Pöschl J., Frommhold D., Hudalla H. (2019). Dystrophin deficiency promotes leukocyte recruitment in mdx mice. Pediatr. Res..

[27] Signorelli M., Ayoglu B., Johansson C., Lochmüller H., Straub V., Muntoni F., Niks E., Tsonaka R., Persson A., Aartsma-Rus A. (2019). Longitudinal serum biomarker screening identifies malate dehydrogenase 2 as candidate prognostic biomarker for Duchenne muscular dystrophy. J. Cachex Sarcopenia Muscle.

[28] Lu H., Huang D., Ransohoff R.M., Zhou L. (2011). Acute skeletal muscle injury: CCL2 expression by both monocytes and injured muscle is required for repair. FASEB J..

[29] Pescatori M., Broccolini A., Minetti C., Bertini E., Bruno C., D’Amico A., Bernardini C., Mirabella M., Silvestri G., Giglio V. (2007). Gene expression profiling in the early phases of DMD: A constant molecular signature characterizes DMD muscle from early postnatal life throughout disease progression. FASEB J..

[30] Starosta A., Konieczny P. (2021). Therapeutic aspects of cell signaling and communication in Duchenne muscular dystrophy. Cell. Mol. Life Sci..

[31] Tawalbeh S.M., Marin W., Morgan G.A., Dang U.J., Hathout Y., Pachman L.M. (2020). Serum protein biomarkers for juvenile dermatomyositis: A pilot study. BMC Rheumatol..

